# The Association of Perfluoroalkyl Substance Exposure and a Serum Liver Function Marker in Korean Adults

**DOI:** 10.3390/toxics11120965

**Published:** 2023-11-28

**Authors:** Jisuk Yun, Soon-Chan Kwon

**Affiliations:** Department of Occupational and Environmental Medicine, Soonchunhyang University Cheonan Hospital, Cheonan 31151, Republic of Korea; sfzcc@naver.com

**Keywords:** PFAS, environmental pollutant, AST, ALT, GGT, KoNEHS, adult

## Abstract

Perfluoroalkyl substances (PFAS), widely used throughout industry and daily life, are currently one of the environmental pollutants garnering the most attention worldwide. Recently, environmental pollutants have had a high profile as one of the main causes of chronic liver disease, such as non-alcoholic fatty liver disease. Research on PFAS is actively underway. Although Korea has a remarkably high prevalence of chronic liver disease, and it continues to increase, only a few studies have revealed the relationship between PFAS and liver disease. In addition, regulations on PFAS in Korea are delayed compared to developed countries, such as Europe and the United States, and public interest is insufficient compared to others. Therefore, we would like to investigate the exposure of Koreans to PFAS in the blood and examine the relationship between these substances and markers of liver function (AST, ALT, and GGT). This study was based on the results of the Korean National Environmental Health Survey (KoNEHS) 2018–2020 (Cycle 4), and a total of 2961 subjects were selected. The concentration of PFAS in the blood of Korean adults was measured to be significantly higher based on the geometric mean compared to the results of recently investigated American adults based on the National Health and Nutrition Examination Survey (NHANES, 2017–2018). A multivariable linear regression analysis adjusted for age, sex, body mass index (BMI), smoking status, alcohol intake, and regular exercise was performed to examine changes in three liver function markers as the serum PFAS concentration increased. We found that some of the five PFAS (PFOA, PFOS, PFHxS, PFNA, and PFDeA) were significantly associated with increased liver enzymes. It is necessary to recognize the threat of PFAS to the human body and to discuss regulations and alternatives in earnest. Continuous follow-up studies are required through a well-designed cohort.

## 1. Introduction

Presently, perfluoroalkyl substances (PFAS), one of the environmental pollutants, have garnered the most attention worldwide. PFAS are widely used in industries and throughout daily life. Due to the presence of carbon–fluorine bonds in PFAS, it has a long half-life and can have adverse effects on several systems in the human body, such as the immune system, endocrine system, reproductive system, etc., [1]. Therefore, perfluorooctansulfonate (PFOS) and perfluorooctanoic acid (PFOA) have been listed as persistent organic pollutants in 2009 and 2019, respectively [2]. Many countries are restricting the production and use of PFAS to minimize their effects on health [3]. However, unlike the developed regions such as Europe and the United States, the regulatory measures in Korea are time-consuming. Due to the nature of their industrial application, PFAS are used in the automobile and semiconductor industries; therefore, their continuous exposure is inevitable. Furthermore, according to a report by the Ministry of Food and Drug Safety of the Republic of Korea in 2022, a study on the risks of PFAS reported that PFAS are unlikely to affect the human body [4].

Over time, many studies have been conducted on the various effects of PFAS on the human body. In particular, we paid attention to the association with the liver and noted that PFAS cause damage to the liver and are associated with liver disease [5]. A meta-analysis by Costello et al. showed that the exact mechanism underlying PFAS hepatotoxicity is still unclear; however, various types of PFAS have exhibited a positive correlation with increased liver function markers, and there is notable experimental evidence from rodent studies that support it [6]. Recently, many studies have shown the relationship between PFAS and non-alcoholic fatty liver disease (NAFLD) [5,7,8], which can progress into severe liver disease, including hepatocellular carcinoma [9,10,11]. The Korean population has a considerably high prevalence of chronic liver disease; therefore, examining the relationship between PFAS and liver disease markers in the Korean population is of great significance [12].

Studies on the various effects of PFAS on the human body are actively underway worldwide, but no large-scale national epidemiological surveys have been carried out in Korea; therefore, there have been many difficulties in epidemiological research. However, recently, for the first time in Korea, a study was conducted correlating serum the concentration of PFAS with the liver function markers based on the Korean National Environmental Health Survey (KoNEHS) 2015–2017 (Cycle 3) carried out by the National Institute of Environmental Research (NIER) [13]. Although this study revealed notable results regarding the correlation between PFAS and liver function markers for the first time in the Korean population, it cannot be considered a representative result because of the relatively smaller sample size of 1404 participants and considering that the data on PFAS at that time were owned by only certain institutions. The most recent epidemiological study, KoNEHS Cycle 4, officially conducted an epidemiological survey of serum concentration of PFAS in the Korean population from 2018 to 2020. The findings of this survey presented the first epidemiological data that can be representative of the concentration of PFAS in the blood of Korean adults.

Based on the findings of this epidemiological survey, this study investigated the changes in aspartate aminotransferase (AST), alanine aminotransferase (ALT), and gamma–glutamyl transferase (GGT), the representative liver function markers. Their levels changed with the increase in the concentration of PFAS in the blood of Korean adults. Semiconductor and automobile manufacturing are the core industries of South Korea, and PFAS are used in major processes in these industries [3]. However, despite the continued regulation of PFAS and discussions on their risks regarding human health worldwide, studies on their effects on the Korean population and discussions on their alternatives are lacking; therefore, this study may provide a theoretical basis and primary data for establishing national policies on PFAS in the future.

## 2. Materials and Methods

### 2.1. Study Population

In total, 4239 adults participated in the KoNEHS carried out by the NIER from 2018 to 2020. On the scheduled day of the survey, a field team consisting of survey experts and sample-collection personnel visited the survey sites and collected a questionnaire, physical measurement data, and biological samples (such as blood and urine). The survey was conducted using a one-on-one interview method by a survey specialist based on the KoNEHS Cycle 4 questionnaire. However, in 2020, face-to-face interviews were not conducted due to the coronavirus disease (COVID-19) outbreak, and physical measurements and blood sample collection were excluded. All participants who were suffering from liver diseases such as hepatitis, cirrhosis, and liver cancer (39 participants) and those with missing survey and blood test information (1239 participants) were excluded from the study. During this survey period, numerous missing values occurred due to the COVID-19 outbreak. Finally, 2961 participants were selected as study subjects.

### 2.2. Liver Function Test

Herein, ALT, AST, and GGT were used as liver function markers. AST and ALT are representative markers of hepatocellular injury and are involved in various metabolic processes in the human body. They are both expressed in various organs other than the liver; ALT is considered more sensitive to liver disease than AST [14]. Both enzymes exhibit higher expression in men than in women [15] and are well known to be closely related to obesity [16]. GGT primarily catalyzes the transfer of a gamma–glutamyl group from peptides to other amino acids; it is upregulated in various hepatobiliary diseases such as alcoholic hepatitis and cirrhosis and is also closely related to obesity [14].

Blood collection was performed through a vein by trained experts dispatched to the investigation area. The collected samples were immediately stored in a tube mixed with anticoagulants, centrifuged at 3000 rpm for 10 min, stored in a cold room (2 to 6 °C), and transferred to a sample management institution (the National Institute of Environmental Research or designated institution) within 24 h for analysis. All three markers were measured in the serum, a centrifuged supernatant. The ADVIA 1800 Auto Analyzer (Siemens Medical Solutions Diagnostics, Los Angeles, CA, USA) was used for analysis. The analytical range was 4.2–1000 U/L and 3.4–1100 U/L for AST and ALT, respectively, and 2.4–1200 U/L for GGT. Additional information on the sample analysis method can be found in the NIER research data [17].

### 2.3. PFAS

In total, five types of PFAS investigated in Cycle 4 of KoNEHS were selected as research subjects. The compounds included in this study were PFOA, PFOS, perfluorohexanesulfonic acid (PFHxS), perfluorononanoic acid (PFNA), and perfluorodecanoic acid (PFDeA), which are the most commonly known PFAS.

Blood samples for PFAS concentration measurements were frozen at −70 °C until analysis. The pre-processing process for analysis is as follows. (i) First, 200 μL of the sample for analysis is placed in a tube for centrifugation made of polypropylene. (ii) Then, 10 μL of internal standard material and 800 μL of acetonitrile was added to the dispensed sample and stirred for about 10 s to denature the protein in the serum, centrifuged at 13,000 rpm for 10 min to precipitate all the protein and dispensed 800 μL of supernatant. (iii) The dispensed sample was concentrated using nitrogen gas for 15–20 min until it did not volatilize, then it was re-dissolved with 50% methanol 25 μL and used as an analytical sample. A high-performance liquid chromatograph/mass spectrometer (HPLC-MS/MS) of Q-Sight Triple Quad (Perkin-Elmer, Waltham, MA, USA) was used for analysis. The test method showed that the reporting limit of detection of perfluorinated compounds in serum was as follows: PFOA was 0.050 μg/L, PFOS was 0.056 μg/L, PFHxS was 0.071 μg/L, PFNA was 0.019 μg/L, and PFDeA was 0.017 μg/L. Further detailed information on the sample analysis method is shown in the NIER research data [17].

### 2.4. Effect of Potential Confounders

We considered age, sex, body mass index (BMI), smoking status, alcohol intake, and regular exercise as potential confounders to determine the association between serum PFAS concentration and liver function markers. The age was based on the date of the survey, and BMI was directly measured by the survey team. The smoking status, alcohol intake, and regular exercise variables were based on a questionnaire prepared for the KoNEHS Cycle 4. The age of the subjects ranged from 19 to 82 years, which was categorized into the following six categories: 19 to 29 years, 30 to 39 years, 40 to 49 years, 50 to 59 years, 60 to 69 years, and 70 years or older. The smoking status was categorized as “non-smoker”, “former”, and “current”. The drinking variable was classified as “Yes” for males with seven or more soju three times a week and for females with five or more soju three times a week. Otherwise, the variable was classified as “No” for both for a lower frequency. In the case of regular exercise, if the participant exercised for more than 30 min three times a week, it was classified as “Yes” and the rest as “No”. BMI was classified as less than 25 kg/m^2^ and 25 kg/m^2^ or more as a reference point based on the WHO recommendations [18].

### 2.5. Statistical Analysis

We determined the number and percentage of the general characteristics of the study subjects. Furthermore, based on the characteristics of the study subjects, the median values and IQR values of the three types of liver function markers and five types of PFAS are shown.

We performed a linear regression analysis to investigate the correlation between PFAS and the liver function marker. Because individual PFAS concentrations and liver function markers are independent and dependent variables, respectively, and they were skewed, they were transformed using a natural logarithm. In the regression analysis, the following two analyses were performed: simple linear regression without correction of the confounder and multivariable linear regression that contained a total of six variables: age, sex, BMI, smoking status, alcohol intake, and regular exercise. Regression analysis was performed using the entire study population and stratified based on sex and BMI. The results of the linear regression analysis were expressed using beta coefficient values. These values were converted to graphics for ease of understanding and shown once again. All statistical analyses used in this study were performed using the R software (R software version 4.2.2). A *p*-value of <0.05 was considered statistically significant.

## 3. Results

Table 1 displays the general characteristics of the study subjects. The mean ± standard deviation (SD) of the age of the study subjects was 52.45 ± 14.74 years; the mean for men was 52.94 ± 15.14 years, whereas the mean for women was 52.08 ± 14.42 years. Subjects over 60 years old represented the highest proportion. Regarding smoking status, “Former” accounted for the largest proportion of men at 42.6%, whereas in the case of women, “Never”, the majority, accounted for the largest proportion with 94.6%. Regarding alcohol intake and regular exercise, “No” accounted for the higher proportion in both men and women. In the case of BMI, a higher proportion of men had a BMI of ≥25 (54.1%), while a higher proportion of women had a BMI of <25 (58%).

The median and IQR measurements results for blood concentrations of the three liver function markers and five PFAS according to each variable, categorized by sex, are presented in Table 2 and Table 3. The levels of the three types of liver function markers based on each variable were differentially distributed. Notably, the median value of each variable was higher for men than for women in all three types of liver function markers, whereas the median value of all three types of liver function markers was higher in the group with a BMI of 25 kg/m^2^ and increased for both men and women. The median concentration of the five types of PFAS based on each variable generally exhibited a tendency to increase with age for both men and women in the case of the age variable. This was highest among subjects in their 60s, who exhibited a decreasing trend among those in their 70s. Furthermore, except for PFDeA in men, the median concentration of the five types of PFAS was highest in the group with a BMI of 25 kg/m^2^ or more for both men and women. The distribution of PFAS concentration according to the variables was additionally presented as Supplementary Data in boxplots to be understood at a glance (Figure S1).

The results of the linear regression analysis are shown in Table 4. When the entire study population was considered, simple linear regression indicated a significant positive association among all five PFAS types and three liver enzymes. In the case of multivariable linear regression adjusted by age, sex, BMI, smoking status, alcohol intake, and regular exposure, it showed a statistically positive correlation, except for GGT and PFDeA.

**Figure 1:**
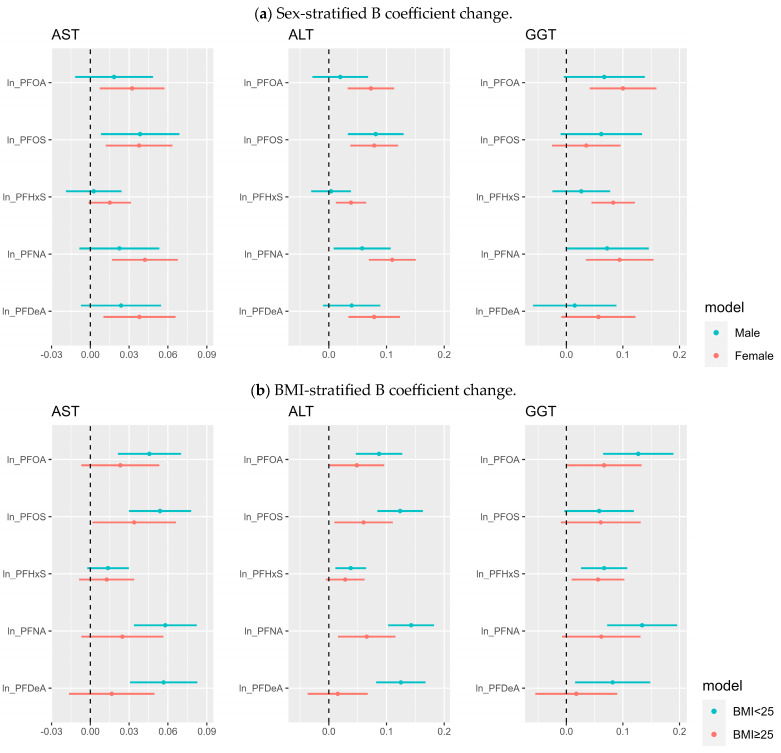
Beta coefficient change in each liver enzyme according to each PFAS obtained using multivariable linear regression, stratified by (**a**) sex and (**b**) BMI.

Regarding sex-stratified analysis, in women, all three liver enzymes and five types of PFAS exhibited a strong positive correlation in simple linear regression. However, AST exhibited a positive correlation with PFOA, PFOS, PFNA, and PFDeA in men. There was no significant association with ALT. However, all five types of PFAS showed a statistically significant correlation with GGT. Multivariable linear regression showed that men showed significant positive associations with PFOS for AST, PFOS, and PFNA for ALT, whereas female showed significant positive associations with PFOA, PFOS, PFNA, and PFDeA for AST, and all five types of PFAS for ALT, and PFOA, PFHxS, and PFNA for GGT. The BMI stratified analysis showed that when BMI was less than 25, multivariable linear regression indicated a significant positive association in all cases except AST and PFHxS and GGT and PFOS. When BMI was 25 or more, multivariable linear regression exhibited a positive association between AST and PFOS, ALT and PFOA, PFOS, PFNA, and GGT with PFOA and PFHxS. Sex and BMI stratified analysis are shown graphically in Figure 1; therefore, they can be easily identified.

**Figure 2:**
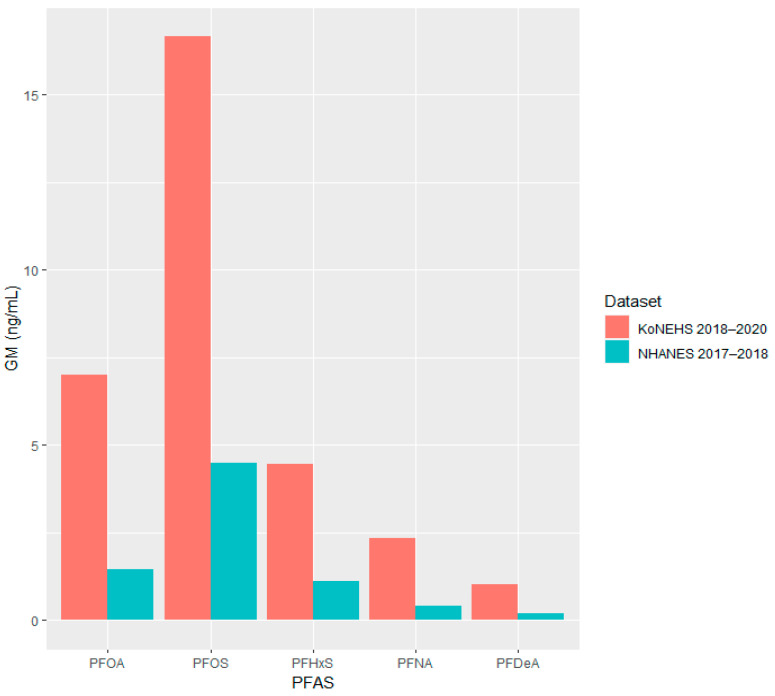
Serum PFAS concentration (geometric mean).

Table 5 shows the geometric mean of serum PFAS concentrations, which were compared using the results of the KoNEHS conducted during 2018–2020 and the most recent NHANES results of the U.S. National Health and Nutrition Survey conducted during 2017–2018. When comparing the serum PFAS concentration of Korean adults with American adults, based on the geometric mean, it was found that the serum PFAS concentration for each substance in Koreans was three to five times higher than that of Americans. In order to see this at a glance, it is additionally presented in a bar graph in Figure 2.

## 4. Discussion

The liver plays a significant role in metabolism, digestion, detoxification, and elimination of substances from the body. Increased liver enzymes are associated with different types of liver disease [14]. PFAS are associated with chronic liver diseases, such as NAFLD and liver cancer [7,8,9], and AST, ALT, and GGT are widely known as biomarkers of these diseases [19]. Therefore, research on the relationship between PFAS and these markers is crucial.

In this study, data representing the general population of Korea were used. We found that some of the five PFAS (namely PFOA, PFOS, PFHxS, PFNA, and PFDeA) were significantly associated with increased liver enzymes. Specifically, a significant positive association was found between more types of PFAS and a wide range of liver function markers in women than in men. Furthermore, most of the B coefficient values were higher in women than men.

Many previous studies have identified the relationship between PFAS and liver function markers. In 2022, Costello et al. performed a meta-analysis on 24 epidemiological studies and reported the relationship between various types of PFAS and liver function markers. Although the results of the individual studies included in Costello’s analysis are inconsistent, the meta-analysis revealed that higher ALT levels are associated with PFOA, PFOS, and PFNA exposure. Based on an individual PFAS perspective, PFOA is associated with higher AST and GGT levels in humans [6]. Along with recent studies, Xiuqi Ma et al. used data from the National Health and Nutrition Examination Survey (NHANES, 2003–2016) and reported that PFOA, PFHxS, and PFNA are positively associated with high levels of ALT [20]. Studies based on the NHANES (2017–2018) revealed a significant positive association between PFOA and GGT, PFHxS and AST, and PFNA and GGT [8]. Two studies of the Canadian population reported a positive association between different types of PFAS and liver function markers [21,22].

Apart from the present study, only a few other studies have suggested that the relationship between serum PFAS concentration and the liver exhibits more adverse effects on female adults. Moreover, the sex-based variation in this effect remains controversial and warrants further investigation. A study by Attanasio et al. on adolescents reported that females are more likely to be susceptible to PFAS hepatotoxicity than males [23]. Furthermore, Nikos Stratakis et al. reported that prenatal exposure is associated with liver injury in girls than in boys [24]. Recently, many studies have shown that PFAS are associated with NAFLD. This association varied with sex, particularly in the case of PFOA and PFNA, which exhibited a positive association with NAFLD in women [25]. Generally, liver metabolic processes often exhibit gender-based variation [26,27]. In vivo experiments exhibit that females are more prone to liver injury when exposed to PFAS than males [7,28]. Hence, the results of this study are acceptable.

In this study, the association between more types of PFAS and liver function indicators was significant and exhibited a stronger effect in the non-obese group than in the obese group. Interestingly, this finding was reported by another Korean study following the study results by Kim et al. based on Cycle 3 of KoNEHS [13]. These results are not consistent with those of previous studies, which showed that the relationship between PFAS and liver function markers is distinct in individuals with obesity [29,30,31]. However, one study showed that alkaline phosphatase (ALP), also a known liver function marker, showed a significantly positive association with PFHxS in non-obese populations [22]. However, ALP was not included in this study because it was not included in the survey of KoNEHS Cycle 4.

Generally, obesity can increase lipid accumulation in the liver and is a known risk factor for NAFLD [32]. It remains unclear whether these results are affected by the inherent limitations of cross-sectional studies, differences in covariate selection, or other factors that are considered a characteristic specific to Korea. In contrast to previous studies that primarily focused on foreigners, few studies determined the association between serum PFAS concentration and liver function markers in Asian adults. Even though similar conclusions were drawn in the previous studies on Asians, they could not be compared with the findings of this study because they did not analyze results based on sex or BMI [33,34].

The exact mechanism underlying perfluorinated compound effects on the liver is yet to be identified. The most widely known mechanism states that PFAS affect the peroxisome proliferator-activated receptor alpha and other receptors (that regulate lipid and glucose metabolisms in the liver and adipocytes), inducing liver inflammation and accumulation of TG [35]; this mechanism has been proven both in vitro and in various animal studies [36,37]. Additionally, a study showed that PFOA and PFOS decreased the level of hepatocyte nuclear factor 4α, a key factor involved in hepatocyte-specific functions, resulting in chronic liver disease [38,39]. Furthermore, some studies have reported PFAS-induced liver damage through different mechanisms, including the activation of nuclear factor-κB involved in inflammation-associated signaling pathways [40], oxidative stress signaling pathways [41], and autophagy [42]. However, consistent results have not been obtained, which warrants the need for further studies.

There are many studies on the effects of PFAS on the liver; however, notably, the results of in vitro experimental studies on PFAS do not necessarily correlate with those of in vivo studies [37], and the presence of liver disease does not necessarily mean that liver function markers are upregulated [43]. In some animal experiments, PFAS-induced histological alterations were not necessarily associated with upregulated liver function markers [44,45]; therefore, extended interpretations based solely on upregulated liver enzymes must be approached very cautiously.

The mortality rate due to liver cancer in Korea is the highest among the Organization for Economic Co-operation and Development countries, and the prevalence of chronic liver disease is also ranked high and continues to increase [46,47]. Additionally, the geometric mean of the serum concentration of PFAS was considerably higher than that of American adults, ranging from three to five times for each substance (Table 5, Figure 2). Environmental pollutants, including PFAS, are widely known to be the tangible risk factor for chronic liver diseases such as NAFLD [48], suggesting that the high prevalence of chronic liver disease in Korea may also be related to the high serum PFAS levels. South Korea is expected to inevitably have a high PFAS exposure because the semiconductor and automobile manufacturing industries make up most of the industrial structure, and perfluorinated compounds are used abundantly. Despite the recent global movement to remove perfluorinated compounds, it has yet to pace this trend. To date, no large-scale epidemiological studies have investigated the correlation between PFAS and liver function markers in the Korean population except for Kim et al. [13] based on the KoNEHS Cycle 3 and the present study. Because this study includes a larger sample size than previous studies and is based on public data, it enhances the reliability of the research results and can garner public interest. More epidemiological studies on the risks of PFAS on human health are warranted in the future for better and consistent results.

This study has some limitations. First, although previous studies have shown that PFAS can act as a causative agent of hepatotoxicity, considering that the present study is cross-sectional, caution is required in interpreting the study results. Second, due to the characteristics of large-scale epidemiological studies, which include only limited experiments, information on biomarkers other than AST, ALT, and GGT that have been included in this study is limited, and information on histology and imaging results is also lacking, making it difficult to interpret the data related to the liver disease. Third, this study investigated the disease history of the subjects based on a questionnaire prepared in advance. Despite the high prevalence of chronic liver disease in Korea, only 39 people were excluded from the study who were previously diagnosed with liver disease. Therefore, it is possible that the actual number of patients with liver disease was greatly underestimated during the study’s subject-selection process.

However, despite these limitations, this study has the following strengths: First, this is one of the few large-scale epidemiological studies examining the relationship between PFAS and liver enzymes, which has been mostly limited to the developed regions, especially the United States. Additionally, this study was conducted on a larger sample size than the study by Kim et al. [13], which was the first to include the Korean population, and was based on the official public data provided by the country, which contributed to the reliability of the results of this study. Second, compared with other developed countries, Korea has a very high and increasing prevalence of chronic liver disease. Therefore, if follow-up studies are conducted consistently because serum PFAS concentrations are also very high, it may provide information regarding the etiology of the highly prevalent chronic liver disease in the Korean population.

This study showed that PFAS were associated with an increase in liver function markers. Considering that the serum concentration of PFAS in Korean adults is very high compared to that of the United States and that continuous exposure is inevitable due to the industrial structure of Korea, it is clear that the public’s awareness should be raised further. It is also thought that serious discussions will be needed to build a national cohort. This study is expected to be used as basic data when identifying and analyzing the health threats of PFAS and as a basis for establishing health policies.

## 5. Conclusions

We found that serum PFAS concentrations were positively correlated with some liver function markers, such as AST, ALT, and GGT. However, this result differs according to sex, obesity, and type of PFAS. In the case of women, the results were generally more sensitive to exposure to most PFAS than men. In addition, the non-obese group generally showed more sensitive results to exposure to most PFAS. Long-term, well-designed longitudinal studies and continuous follow-up are needed.

## Figures and Tables

**Table 1:** General characteristics of the study subjects.

Characteristics	Total	Men	Women
N	2961	1279	1682
Age			
19–29	238 (8.0)	102 (8.0)	136 (8.1)
30–39	393 (13.3)	169 (13.2)	224 (13.3)
40–49	573 (19.4)	247 (19.3)	326 (19.4)
50–59	650 (22.0)	250 (19.5)	400 (23.8)
60–69	719 (24.3)	319 (24.9)	400 (23.8)
70	388 (13.1)	192 (15.0)	196 (11.7)
Mean age	52.45 ± 14.74	52.94 ± 15.14	52.08 ± 14.42
Smoking status			
Never	1913 (64.6)	321 (25.1)	1592 (94.6)
Former	589 (19.9)	545 (42.6)	44 (2.6)
Current	459 (15.5)	413 (32.3)	46 (2.7)
Alcohol intake ^a^			
No	2655 (89.7)	1034 (80.8)	1621 (96.4)
Yes	306 (10.3)	245 (19.2)	61 (3.6)
Regular exercise ^b^			
No	2062 (69.6)	890 (69.6)	1172 (69.7)
Yes	899 (30.4)	389 (30.4)	510 (30.3)
BMI (kg/m^2^)			
<25	1563 (52.8)	587 (45.9)	976 (58.0)
≥25	1398 (47.2)	692 (54.1)	706 (42.0)

Values are presented as the number (%) or mean (±standard deviation). BMI: body mass index. ^a^ Alcohol intake was considered “Yes” for males who drank seven or more soju three times a week or “No” for females who drank five or more soju three times a week. ^b^ Regular exercise was considered “Yes” for individuals who exercised for >30 min three times a week; otherwise, “No”.

**Table 2:** Distribution of the median (IQR) values of serum liver function marker concentration in the study population according to the variables.

	AST (U/L)		ALT (U/L)		GGT (U/L)	
Characteristics	Men	Women	Men	Women	Men	Women
Age						
19–29	23 (20, 29)	20 (18, 23)	23.5 (17, 42)	14 (12, 17.5)	22 (15, 37)	11.5 (9, 16)
30–39	25 (21, 31)	21 (18, 24)	29 (20, 48)	16 (13, 22.5)	30 (18, 53)	13 (10, 19)
40–49	26 (22, 30	21 (19, 24)	28 (20, 39)	16 (13, 21)	33 (22, 50.5)	13 (10, 21)
50–59	26 (22, 29)	24 (21, 28)	25 (20, 32)	20 (16, 27)	32.5 (21, 59)	17 (12, 27)
60–69	27 (23, 31)	25 (22, 29)	25 (19, 31)	21 (17, 27)	29 (20, 47)	17 (12, 24)
>70	26 (22, 30)	25 (22, 30)	21.5 (17, 30)	20 (16, 25)	24 (17, 38)	16 (12, 24.5)
Smoking status						
Never	25 (22, 29)	23 (22, 27)	25 (20, 35)	19 (15, 25)	25 (17, 38)	15 (11, 23)
Former	26 (23, 31)	22.5 (19, 26.5)	25 (19, 34)	17 (14, 25.5)	29 (19, 47)	14 (10, 20)
Current	25 (21, 31)	22 (19, 24)	25 (18, 35)	17 (14, 21)	34 (22, 60)	20.5 (13, 33)
Alcohol intake ^a^						
No	25 (22, 30)	23 (20, 27)	25 (19, 34)	19 (15, 25)	26 (18, 42)	15 (11, 23)
Yes	27 (24, 33)	22 (19, 25)	26 (19, 36)	16 (15, 23)	49 (30, 97)	18 (12, 33)
Regular exercise ^b^						
No	26 (22, 31)	23 (20, 27)	26 (19, 36)	18 (14, 25)	30 (20, 53)	15 (11, 23)
Yes	26 (22, 29)	24 (21, 27)	24 (18, 31)	19 (15, 25)	26 (17, 41)	14 (11, 22)
BMI (kg/m^2^)						
<25	25 (21, 29)	22 (20, 26)	22 (17, 29)	16 (13, 22)	25 (17, 42)	14 (10, 19)
≥25	26 (23, 32)	24 (21, 28)	28 (22, 40)	21 (17, 28)	33 (22, 55.5)	18 (13, 28)

AST, aspartate aminotransferase; ALT, alanine transaminase; BMI, body mass index; GGT, gamma–glutamyl transferase; ^a^ Alcohol intake was considered “Yes” for males who drank seven or more soju three times a week or “No” for females who drank five or more soju three times a week. ^b^ Regular exercise was considered “Yes” for individuals who exercised for >30 min three times a week; otherwise, “No”.

**Table 3:** Distribution of the median (IQR) values of serum PFAS concentration in the study population according to the variables.

	PFOA (μg/L)		PFOS (μg/L)		PFHxS (μg/L)	
Characteristics	Men	Women	Men	Women	Men	Women
Age						
19–29	4.24 (3.05, 5.58)	3.74 (2.70, 5.00)	8.96 (6.50, 11.78)	7.19 (5.04, 10.06)	2.79 (2.08, 4.02)	1.83 (1.31, 2.66)
30–39	6.24 (4.40, 8.05)	4.33 (3.24, 5.70)	12.64 (9.02, 17.17)	9.74 (7.28, 13.26)	4.08 (2.93, 5.81)	2.45 (1.69, 3.96)
40–49	7.17 (5.34, 9.50)	5.26 (3.96, 7.32)	14.57 (10.89, 19.35)	11.82 (8.47, 15.83)	4.71 (3.32, 6.88)	3.04 (2.03, 4.53)
50–59	8.85 (6.16, 11.95)	8.01 (5.90, 11.62)	19.09 (14.75, 26.89)	18.77 (13.97, 24.61)	5.77 (3.82, 9.67)	4.44 (3.08, 7.07)
60–69	8.69 (6.44, 12.71)	9.00 (6.61, 12.37)	25.80 (17.62, 34.02)	23.55 (17.72, 30.81)	5.50 (3.81, 8.10)	5.38 (3.52, 7.32)
>70	8.23 (5.86, 11.07)	8.32 (6.40, 11.70)	25.01 (17.57, 33.28)	22.87 (16.92, 30.68)	5.34 (3.58, 8.44)	5.24 (3.08, 8.20)
Smoking status						
Never	6.85 (4.65, 9.32)	6.82 (4.59, 9.98)	16.61 (10.81, 25.68)	16.71 (10.55, 24.19)	4.25 (2.80, 6.87)	3.89 (2.41, 6.36)
Former	8.23 (5.92, 11.49)	5.13 (3.68, 7.36)	20.71 (14.71, 29.61)	11.73 (6.93, 16.19)	5.38 (3.52, 8.30)	2.96 (2.23, 6.37)
Current	7.13 (5.14, 9.63)	5.71 (3.89, 7.52)	15.15 (10.79, 24.43)	12.04 (6.38, 18.61)	4.95 (3.40, 7.38)	3.73 (2.23, 5.63)
Alcohol intake ^a^						
No	7.39 (5.12, 10.31)	6.75 (4.58, 9.87)	17.62 (11.96, 27.09)	16.58 (10.48, 24.11)	4.80 (3.18, 7.64)	3.88 (2.42, 6.31)
Yes	8.16 (6.12, 11.31)	5.76 (3.73, 7.66)	18.99 (13.51, 28.19)	11.41 (6.74, 16.44)	5.45 (3.55, 8.15)	3.49 (1.88, 6.84)
Regular exercise ^b^						
No	7.35 (5.16, 10.16)	6.41 (4.28, 9.19)	17.01 (11.54, 26.43)	15.38 (9.73, 22.73)	4.84 (3.21, 7.38)	3.70 (2.28, 6.13)
Yes	7.90 (5.71, 11.03)	7.83 (5.13, 11.43)	20.26 (14.36, 28.61)	18.64 (12.37, 26.41)	5.36 (3.41, 8.38)	4.13 (2.69, 6.68)
BMI (kg/m^2^)						
<25	7.26 (5.34, 10.27)	6.34 (4.30, 9.31)	17.66 (12.16, 26.61)	15.05 (9.70, 22.40)	4.86 (3.15, 7.61)	3.53 (2.22, 6.10)
≥25	7.82 (5.32, 10.77)	7.41 (4.92, 11.03)	18.02 (12.24, 27.84)	18.59 (12.01, 26.41)	5.01 (3.38, 7.90)	4.15 (2.70, 6.60)
	PFNA (μg/L)		PFDeA (μg/L)			
Characteristics	Men	Women	Men	Women		
Age						
19–29	1.19 (0.88, 1.56)	1.03 (0.75, 1.33)	0.52 (0.41, 0.70)	0.52 (0.40, 0.70)		
30–39	1.81 (1.30, 2.43)	1.26 (0.92, 1.70)	0.78 (0.58, 1.04)	0.63 (0.51, 0.83)		
40–49	2.24 (1.66, 3.02)	1.61 (1.17, 2.41)	0.90 (0.67, 1.27)	0.76 (0.60, 1.09)		
50–59	3.16 (2.31, 4.22)	2.76 (2.04, 3.79)	1.31 (0.91, 1.69)	1.14 (0.84, 1.55)		
60–69	3.54 (2.42, 4.91)	3.31 (2.44, 4.49)	1.46 (1.01, 2.13)	1.32 (1.02, 1.72)		
>70	3.12 (2.34, 4.59)	3.30 (2.32, 4.36)	1.38 (1.04, 2.12)	1.25 (0.88, 1.77)		
Smoking status						
Never	2.24 (1.48, 3.28)	2.33 (1.45, 3.52)	0.96 (0.64, 1.38)	0.99 (0.69, 1.43)		
Former	3.06 (2.16, 4.38)	1.66 (1.14, 2.39)	1.27 (0.91, 1.85)	0.75 (0.53, 1.24)		
Current	2.46 (1.66, 3.37)	1.90 (1.17, 2.81)	0.98 (0.66, 1.46)	0.82 (0.60, 1.20)		
Alcohol intake ^a^						
No	2.57 (1.72, 3.72)	2.31 (1.45, 3.50)	1.07 (0.70, 1.55)	0.99 (0.68, 1.42)		
Yes	2.94 (2.18, 4.05)	1.89 (1.02, 3.01)	1.24 (0.85, 1.77)	0.84 (0.56, 1.23)		
Regular exercise ^b^						
No	2.55 (1.72, 3.64)	2.16 (1.30, 3.34)	1.06 (0.70, 1.56)	0.93 (0.64, 1.36)		
Yes	2.85 (1.96, 4.11)	2.57 (1.72, 3.80)	1.19 (0.84, 1.66)	1.08 (0.77, 1.51)		
BMI (kg/m^2^)						
<25	2.58 (1.80, 3.72)	2.09 (1.29, 3.13)	1.12 (0.75, 1.53)	0.93 (0.65, 1.34)		
≥25	2.70 (1.77, 3.92)	2.64 (1.62, 3.91)	1.09 (0.72, 1.64)	1.08 (0.72, 1.52)		

BMI, body mass index; PFOA, perfluorooctanoic acid; PFOS, perfluorooctane sulfonate; PFHxS, perfluorohexane sulfonic acid; PFNA, perfluorononanoic acid; PFDeA, perfluorodecanoate. ^a^ Alcohol intake was considered “Yes” for males who drank seven or more soju three times a week or “No” for females who drank five or more soju three times a week. ^b^ Regular exercise was considered “Yes” for individuals who exercised for >30 min three times a week; otherwise, “No”.

**Table 4:** Linear regression analysis results of beta coefficient changes in the natural log-transformed concentration of each liver function marker according to the increase in the natural log-transformed concentration of each PFAS for the entire study population, stratified by sex and BMI.

Multivariable change in ln AST								
	Total		Men		Women		BMI	
	Crude	Adjusted model ^a^	Crude	Adjusted model ^b^	Crude	Adjusted model ^b^	<25 (n = 1563) ^c^	≥25 (n = 1398) ^c^
lnPFOA	0.09(0.07–0.11) ***	0.04(0.02–0.05) ***	0.04(0.01–0.07) **	0.02(−0.01–0.05)	0.11(0.09–0.13) ***	0.03(0.01–0.06) *	0.05(0.02–0.07) ***	0.02(−0.01–0.05)
lnPFOS	0.10(0.08–0.12) ***	0.04(0.02–0.06) ***	0.05(0.03–0.08) ***	0.04(0.01–0.07) *	0.12(0.10–0.14) ***	0.04(0.01–0.06) **	0.05(0.03–0.08) ***	0.03(0.00–0.07) *
lnPFHxS	0.05(0.04–0.06) ***	0.01(0.00–0.03) *	0.01(−0.01–0.03)	0.00(−0.02–0.02)	0.06(0.04–0.08) ***	0.02(−0.00–0.03)	0.01(−0.00–0.03)	0.01(−0.01–0.03)
lnPFNA	0.10(0.08–0.12) ***	0.04(0.02–0.06) ***	0.05(0.02–0.07) **	0.02(−0.01–0.05)	0.12(0.10–0.14) ***	0.04(0.02–0.07) **	0.06(0.03–0.08) ***	0.02(−0.01–0.06)
lnPFDeA	0.10(0.08–0.11) ***	0.03(0.01–0.05) **	0.05(0.02–0.07) **	0.02(−0.01–0.05)	0.13(0.10–0.15) ***	0.04(0.01–0.07) **	0.06(0.03–0.08) ***	0.02(−0.02–0.05)
B. Multivariable change in ln ALT								
	Crude	Adjusted model ^a^	Crude	Adjusted model ^b^	Crude	Adjusted model	<25 (n = 1563) ^c^	>=25 ^c^
lnPFOA	0.12(0.09–0.15) ***	0.07(0.04–0.10) ***	−0.01(−0.06–0.04)	0.02(−0.03–0.07)	0.17(0.14–0.21) ***	0.07(0.03–0.11) ***	0.09(0.05–0.13) ***	0.05(0.00–0.10) *
lnPFOS	0.13(0.10–0.16) ***	0.09(0.06–0.12) ***	0.00(−0.04–0.05)	0.08(0.03–0.13) **	0.18(0.15–0.22) ***	0.08(0.04–0.12) ***	0.12(0.08–0.16) ***	0.06(0.01–0.11) *
lnPFHxS	0.08(0.06–0.11) ***	0.04(0.01–0.06) **	−0.01(−0.04–0.03)	0.00(−0.03–0.04)	0.10(0.07–0.12) ***	0.04(0.01–0.06) **	0.04(0.01–0.06) **	0.03(−0.01–0.06)
lnPFNA	0.14(0.11–0.17) ***	0.10(0.07–0.13) ***	−0.01(−0.06–0.03)	0.06(0.01–0.11) *	0.20(0.17–0.23) ***	0.11(0.07–0.15) ***	0.14(0.10–0.18) ***	0.07(0.02–0.12) *
lnPFDeA	0.11(0.08–0.14) ***	0.06(0.03–0.10) ***	−0.03(−0.07–0.01)	0.04(−0.01–0.09)	0.19(0.15–0.23) ***	0.08(0.03–0.12) **	0.12(0.08–0.17) ***	0.02(−0.04–0.07)
C. Multivariable change in ln GGT								
	Crude	Adjusted model ^a^	Crude	Adjusted model ^b^	Crude	Adjusted model	<25 (n = 1563) ^c^	>=25 ^c^
lnPFOA	0.21(0.16–0.26) ***	0.10(0.05–0.14) ***	0.13(0.06–0.20) ***	0.07(−0.00–0.14)	0.19(0.14–0.24) ***	0.10(0.04–0.16) **	0.13(0.07–0.19) ***	0.07(0.00–0.13) *
lnPFOS	0.18(0.13–0.22) ***	0.06(0.01–0.10) *	0.07(0.01–0.14) *	0.06(−0.01–0.13)	0.15(0.10–0.19) ***	0.04(−0.03–0.10)	0.06(−0.00–0.12)	0.06(−0.01–0.13)
lnPFHxS	0.17(0.14–0.21) ***	0.06(0.03–0.09) ***	0.06(0.01–0.12) *	0.03(−0.02–0.08)	0.13(0.10–0.17) ***	0.08(0.04–0.12) ***	0.07(0.03–0.11) **	0.06(0.01–0.10) *
lnPFNA	0.23(0.18–0.27) ***	0.10(0.05–0.14) ***	0.07(0.01–0.14) *	0.07(−0.00–0.15)	0.19(0.14–0.23) ***	0.09(0.03–0.15) **	0.13(0.07–0.20) ***	0.06(−0.01–0.13)
lnPFDeA	0.18(0.14–0.23) ***	0.04(−0.01–0.09)	0.13(0.06–0.20) ***	0.01(−0.06–0.09)	0.17(0.12–0.23) ***	0.06(−0.01–0.12)	0.08(0.02–0.15) *	0.02(−0.06–0.09)

Values are presented as numbers (95% confidence interval). AST, aspartate aminotransferase; ALT, alanine transaminase; BMI, body mass index; GGT, gamma–glutamyl transferase; PFOA, perfluorooctanoic acid; PFOS, perfluorooctane sulfonate; PFHxS, perfluorohexane sulfonic acid; PFNA, perfluorononanoic acid; PFDeA, perfluorodecanoate. ^a^ Adjusted for sex, age, smoking status, alcohol intake, regular exercise, and BMI. ^b^ Adjusted for age, smoking status, alcohol intake, regular exercise, and BMI. ^c^ Adjusted for sex, age, smoking status, alcohol intake, and regular exercise.; * *p* < 0.05; ** *p* < 0.01; *** *p* < 0.001

**Table 5:** Serum PFAS concentration (geometric mean, 95% confidence interval).

	NHANES 2017–2018 (n = 1929)	KoNEHS 2018–2020 (n = 2961)
PFOA (ng/mL)	1.45 (1.35–1.56)	7.01 (6.87–7.15)
PFOS (ng/mL)	4.50 (4.15–4.89)	16.67 (16.31–17.04)
PFHxS (ng/mL)	1.11 (1.03–1.21)	4.47 (4.35–4.60)
PFNA (ng/mL)	0.42 (0.37–0.47)	2.36 (2.31–2.42)
PFDeA (ng/mL)	0.20 (0.18–0.22)	1.03 (1.01–1.05)

NHANES, National Health and Nutrition Examination Survey; KoNEHS, Korean National Environmental Health Survey; PFOA, perfluorooctanoic acid; PFOS, perfluorooctane sulfonate; PFHxS, perfluorohexane sulfonic acid; PFNA, perfluorononanoic acid; PFDeA, perfluorodecanoate.

## Data Availability

The data (Korean National Environmental Health Survey, 2018–2020) that have been used in this paper are not openly accessible but can be requested via https://www.nier.go.kr/NIER/index.do (accessed on 22 November 2023).

## Supplementary Materials

The following supporting information can be downloaded at https://www.mdpi.com/2305-6304/11/12/965/s1, Figure S1: Distribution of the median (IQR) values of serum PFAS concentration in the study population according to the variables.
