# Supplementary material for: The Association of Perfluoroalkyl Substance Exposure and a Serum Liver Function Marker in Korean Adults
Source: Toxics. 2023 Nov 28;11(12):965. doi: 10.3390/toxics11120965 (PMC10748130; doi:10.3390/toxics11120965)
Supplement: Supplementary file 1 [file toxics-11-00965-s001.zip › Figure S1 Distribution of the median.pdf]

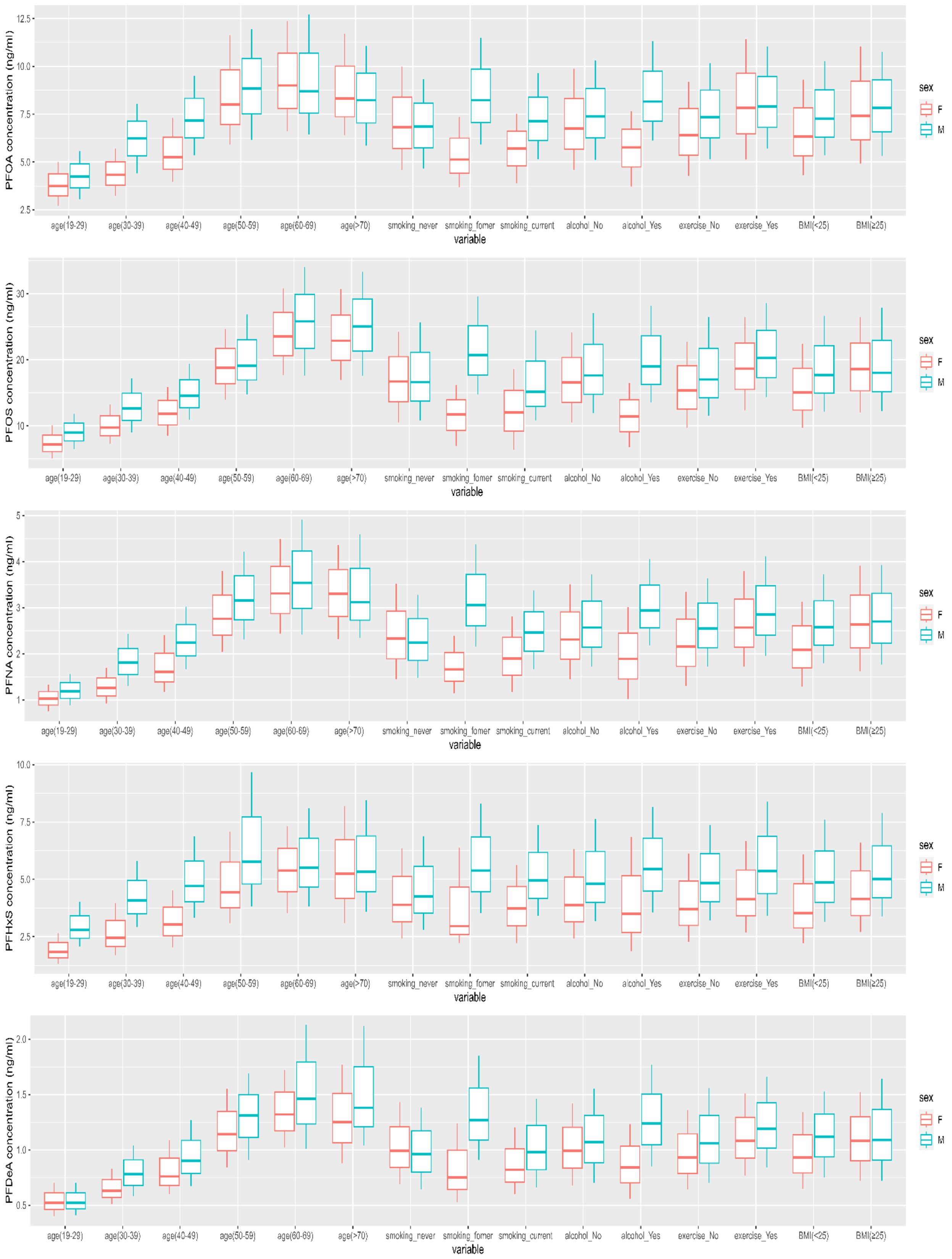

Figure S1 Distribution of the median (IQR) values of serum PFAS concentration in the study population according to the variables.
